# *Salmonella* Typhi acquires diverse plasmids from other Enterobacteriaceae to develop cephalosporin resistance

**DOI:** 10.1016/j.ygeno.2021.05.003

**Published:** 2021-07

**Authors:** Jobin John Jacob, Agila Kumari Pragasam, Karthick Vasudevan, Balaji Veeraraghavan, Gagandeep Kang, Jacob John, Vasant Nagvekar, Ankur Mutreja

**Affiliations:** a Department of Clinical Microbiology, Christian Medical College, Vellore, Tamil Nadu, India; b Division of Gastrointestinal Sciences, Christian Medical College, Vellore, Tamil Nadu, India; c Department of Community Health, Christian Medical College, Vellore, Tamil Nadu, India; d Department of Physician/Internal Medicine, Lilavati Hospital & Research Centre, Mumbai, India; e Department of Medicine, University of Cambridge, Cambridge, United Kingdom

**Keywords:** *Salmonella* Typhi, Typhoid fever, Cephalosporin resistance, XDR, India, H58 lineages

## Abstract

**Background:**

Recent reports have established the emergence and dissemination of extensively drug resistant (XDR) H58 *Salmonella* Typhi clone in Pakistan. In India where typhoid fever is endemic, only sporadic cases of ceftriaxone resistant *S.* Typhi are reported. This study aimed at elucidating the phylogenetic evolutionary framework of ceftriaxone resistant *S.* Typhi isolates from India to predict their potential dissemination.

**Methods:**

Five ceftriaxone resistant *S.* Typhi isolates from three tertiary care hospitals in India were sequenced on an Ion Torrent Personal Genome Machine (PGM). A core genome single-nucleotide-polymorphism (SNP) based phylogeny of the isolates in comparison to the global collection of MDR and XDR *S.* Typhi isolates was built. Two of five isolates were additionally sequenced using Oxford Nanopore MinION to completely characterize the plasmid and understand its transmission dynamics within Enterobacteriaceae.

**Results:**

Comparative genomic analysis and detailed plasmid characterization indicate that while in Pakistan (4.3.1 lineage I) the XDR trait is associated with *bla*_CTX-M-15_ gene on IncY plasmid, in India (4.3.1 lineage II), the ceftriaxone resistance is due to short term persistence of resistance plasmids such as IncX3 (*bla*_SHV-12_) or IncN (*bla*_*T*EM-1B_ + *bla*_DHA-1_).

**Conclusion:**

Considering the selection pressure exerted by the extensive use of ceftriaxone in India, there are potential risks for the occurrence of plasmid transmission events in the predominant H58 lineages. Therefore, continuous monitoring of *S*. Typhi lineages carrying plasmid-mediated cephalosporin resistant genes is vital not just for India but also globally.

## Highlights

• *S*. Typhi to develop cephalosporin resistance by acquiring diverse plasmids from other Enterobacteriaceae.
• Independent acquisition of drug-resistant plasmids such as IncX3 and IncN with genes encoding beta-lactamases in H58 lineage II of *S*. Typhi.
• A short-term persistence of drug-resistant plasmids in H58 lineage II can be the reason for the sporadic cases cephalosporin resistant *S*. Typhi in India.
• Plasmid acquisition and maintenance of cephalosporin resistant *S*. Typhi appears to be specific to the phylogenetic lineage.
• Critical strategies in monitoring and control of cephalosporin resistant *S*. Typhi is needed to tackle further public health crisis.

## 1 Introduction

Enteric fever is a severe systemic infection caused primarily by *Salmonella enterica* serovar Typhi and serovar Paratyphi A/B/C [1]. These serovars are restricted to human hosts and typically occur in low and middle – income countries (LMIC) [2]. Although direct faecal-oral route is the predominant mode of transmission, recent reports suggest that indirect transmission may also occur as the bacteria can survive for extended periods in the environment [3]. The global burden of typhoid fever is estimated to be between 11 and 21 million with 128, 000 to 161, 000 deaths annually [4]. Global data suggests that the majority of the reported enteric fever morbidity and mortality takes place in endemic regions of South Asian, Southeast Asian and African countries [5].

The management of enteric fever is challenging due to the emergence of antibiotic resistant *S.* Typhi strains and their changing resistance profiles [6]. The indiscriminate use of first line antimicrobial agents (ampicillin, chloramphenicol and co-trimoxazole) during the 1960s led to the emergence of multi drug resistance (MDR) in sporadic cases initially, followed by larger outbreaks during 1970–1990 [7]. Fluoroquinolones (FQs) such as ciprofloxacin then became the drug of choice for treatment of MDR *S*. Typhi. Nevertheless, the decreased ciprofloxacin susceptibility (DCS) phenotype became dominant globally within a few years, resulting in clinical failures [8]. Currently, ceftriaxone and azithromycin are the drugs of choice. However, there are reports of ceftriaxone resistant and azithromycin resistant *S*. Typhi [9].

Whole-genome sequencing of globally collected *S*. Typhi has identified a single dominant lineage “H58” (genotype 4.3.1), with regional specific dominance of H58 lineage I (4.3.1.1) in Bangladesh and Pakistan, and H58 lineage II (4.3.1.2) in India and Nepal [10,11]. Following the dominance of MDR and FQ resistant lineages, *S.* Typhi isolates with extensive drug resistance (XDR) have emerged in Sindh, Pakistan, with resistance to ampicillin, chloramphenicol, co-trimoxazole, fluoroquinolones and third-generation cephalosporins [12,13]. This large-scale outbreak reported a total of 5274 XDR *S*. Typhi cases between 2016 and 2018 [14]. The XDR *S*. Typhi isolates carried an IncY plasmid harboring a *bla*_CTX-M-15_ and *qnrS1* gene while the composite transposon antimicrobial resistance (AMR) cassette (*catA1*, *bla*_TEM-1_, *dfrA7*, *sul1* and *sul2*) conferring resistance to first-line drugs was integrated into the chromosome [12]. In India where typhoid fever is endemic, only limited number of ceftriaxone resistant *S.* Typhi have been reported till date [[15], [16], [17]].

Until recently, phylogenetic inferences of the evolution of cephalosporin resistant *S.* Typhi were limited to the reported XDR *S.* Typhi outbreak in Pakistan [12], possibly because only sporadic cases are reported across other locations in South Asia. Since *S.* Typhi H58 lineage can acquire MDR plasmids (IncY) from other Enterobacteriaceae, this event could also occur in other regions of Asia where typhoid is endemic [12]. Our study investigated the phylogenetic relationship of cephalosporin resistance in *S*. Typhi with special reference to the endemic regions in South Asia. We also characterized the plasmid that was seen a potential acquisition from other Enterobacteriaceae to predict the possibility of the rise and spread of cephalosporin resistant *S*. Typhi in India.

## 2 Results

### 2.1 Identification of ceftriaxone resistant *S*. Typhi

All the study isolates were phenotypically and biochemically identified as *S*. Typhi and serologically confirmed by traditional serotyping. The antimicrobial susceptibility profiling of our study isolates showed resistance to ampicillin*,* ceftriaxone and ciprofloxacin whilst being sensitive to chloramphenicol, co-trimoxazole and azithromycin*.* Among the five isolates, none were MDR and all showed similar AST patterns. The demographic details and the resistance profiles of the study isolates are given in Table 1.

**Table 1:** Molecular fingerprint of cephalosporin resistant *S*. Typhi.

Country of origin (Reference)	Location	Isolate Id & Accession No.	Year	Acquired Resistance			Chromosomal resistance (QRDR)	Lineage	Ceftriaxone MIC (μg/ml)
				Beta lactamases	Other AMR determinants	Plasmids			
DRC (22)		ERR1862943	2015	CTX-M-15, TEM-1D	*dfrA7, sul1, aac6’*	IncY	S83F	Non-H58 (2.5.1)	–
Philippines (23)	Travel associated	ERR578472 ERR578473	2012	TEM-1B, SHV-12	*dfrA19, aac6’, aph3’, aph6’, tetD, mcr-9*	IncHI2	Absent	Non-H58 (3)	32 16
Pakistan (12)	Sindh Outbreak	ERR2093236 - ERR2093334	2016–2017	CTX-M-15, TEM-1	*dfrA7, catA1, sul1 and sul2, qnrS, strAB*	IncY	S83F	H58 Lineage I (4.3.1.1P)	>64
Bangladesh (20,21)	Dhaka	ERR2059823	2000	CTX-M-15, TEM-1B	*aac6’*	IncI1	S83Y	Non-H58	>32
India (15–17) *This study	Gurgaon, Haryana (*n* = 2)	Gurgaon01 (ERR3527963)	2019	TEM-1B, CTX-M-15	*dfrA14, sul1/2, tetA, qnrS1, aac6’, aph6’, aph3’*	IncY	S83Y	H58 Lineage I (4.3.1.1)	–
		Gurgaon02 (ERR3527964)	2019		*aac(6”)Iaa, tetA*	IncY (lost while sub culturing)	S83Y	H58 Lineage I (4.3.1.1)	–
	Mumbai* (*n* = 4)	458,426 (MQUO00000000) 430,040 (MQUM00000000) 429,038 (MQUL00000000) LHST_2018 (CP052767 and CP052768)	2016 2018	SHV-12	*qnrB7*	IncX3	S83F, D87N, S80I	H58 Lineage II (4.3.1.2)	16
	Vellore* (*n* = 1)	CMCST_CEPR_1 CP053702 and CP053703	2015	TEM-1B, DHA	*qnrB4, aac6’, sul1*	IncN	S83F, E84K	H58 Lineage II (4.3.1.2)	>16

DRC – Democratic Republic of the Congo.

Genome based MLST analysis classified the isolates to ST1 and identified them as 4.3.1.2 (H58 lineage II) genotype. Resistome analysis from the whole genome revealed the presence of *bla*_SHV-12_, and *qnrB7* genes in four Mumbai isolates, while the Vellore isolate carried *bla*_*T*EM-1B_, *bla*_DHA-1_, *qnrB4*, *aac(6)Iaa*, and *sul1* resistance genes. Ciprofloxacin resistance could be attributed to QRDR triple mutations (*gyrA*: S83F, D87N, *parC*: S80I) for the Mumbai isolates and double mutations (*gyrA*: S83F, *parC*: E84K) for the Vellore isolate.

### 2.2 Ceftriaxone resistant lineages circulating in India

**Fig. 1:**
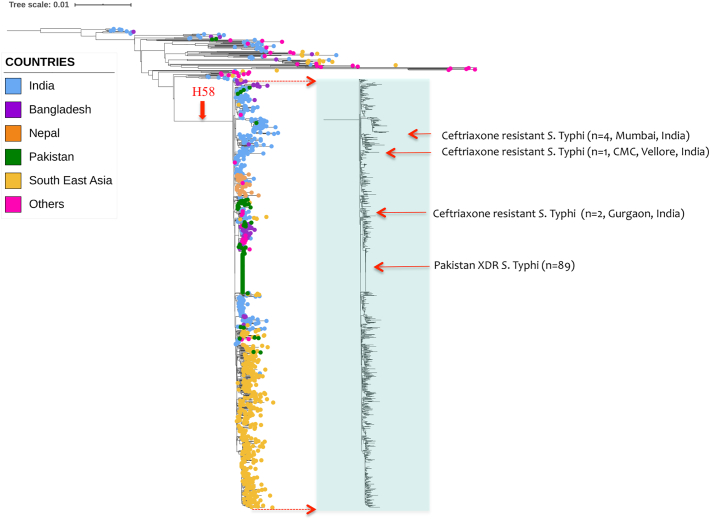
Maximum likelihood tree of 1005 *S.* Typhi (H58 and non—H58) inferred from 2072 SNPs and rooted against the reference genome CT18. Light shaded box indicates H58 population, while the rest are non-H58 group. The tips of the tree are colored according to the geographical origin of the genomes. Red colored squared boxes with arrows indicates the position of ceftriaxone resistant *S.* Typhi in the context of global phylogeny, indicating relatedness with respect to the geographical locations. Tree scale indicates the number of substitutions per genome. (For interpretation of the references to colour in this figure legend, the reader is referred to the web version of this article.)

To investigate the emergence of ceftriaxone resistance in Indian *S*. Typhi isolates, the phylogenetic relationship of study isolates was compared with the representative isolates (H58 and non—H58) from the global *S*. Typhi collection (Fig. 1). Ceftriaxone resistant isolates from the recently reported XDR *S*. Typhi outbreak in the Sindh, Pakistan as well as sporadic ceftriaxone resistant isolates from Gurgaon, India and Dhaka, Bangladesh were included in the phylogenetic tree (Supplementary Table S1). The XDR *S*. Typhi isolates from Pakistan formed a distinct clade (4.3.1.1P) within the H58 lineage. Isolates reported to have originated from a direct travel history from Pakistan were also found to be in the same cluster. The two documented ceftriaxone resistant isolates from Bangladesh clustered with the non-H58 lineage along with the ceftriaxone sensitive isolates from the same location. Interestingly the five study isolates from India did not cluster together, and were distributed across the 4.3.1 lineage II. The four cephalosporin resistant isolates from Mumbai, India formed a sub clade within the H58 lineage II and differed by 2 SNPs from the closest isolates within the subclade. However, the recently reported ceftriaxone resistant *S*. Typhi from Gurgaon, India clustered within the sub clade of H58 lineage I with a difference of 3 SNPs from the ceftriaxone sensitive isolates. This phylogenetic difference, alongside the distinct plasmid mediated mechanistic difference, suggests that all the ceftriaxone resistant *S*. Typhi isolates from India have evolved independently from respective geographical locations.

### 2.3 Non-IncY plasmids carrying ceftriaxone resistance genes

**Fig. 2:**
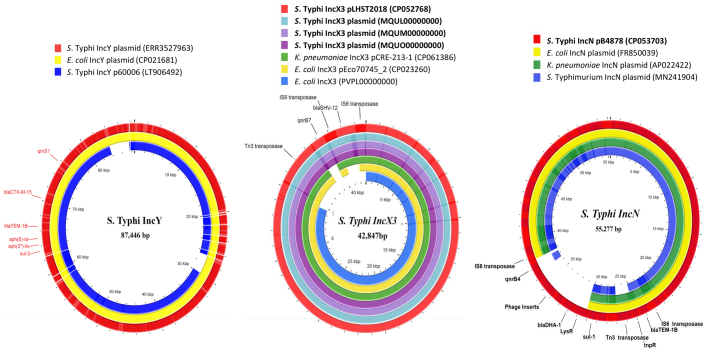
Circular representation of plasmids assembled from *S*. Typhi isolates displayed using CG view server with reference genomes. (a) The IncY plasmid from cephalosporin resistant *S*. Typhi isolate from Gurgaon, India (ERR3527963*) is compared with similar IncY plasmids identified from XDR Pakistan *S*. Typhi (Plasmid p60006: LT906492) and highly similar *E. coli* IncY plasmid (CP021681). (b) Comparison of IncX3 plasmid from cephalosporin resistant Indian *S*. Typhi isolates (*n* = 4) with global Enterobacteriaceae associated IncX3 plasmids (CP061386, CP023260) and IncX3 plasmid from Indian *E. coli* isolate (PVPL00000000*) (c) The IncN plasmid assembled from cephalosporin resistant *S*. Typhi Vellore isolate (CP053703) was compared with representative Enterobacteriaceae associated IncN plasmids (FR850039, AP022422, MN241904 from Genbank.(*Draft genome sequence accession numbers: Plasmids from draft genomes were separated using plasmidSPAdes [38]).

The complete circular plasmids of two representative isolates were used as a reference to reconstruct plasmids from the short-read assembly in other isolates. Four study isolates from Mumbai carried the *bla*_SHV-12_ and *qnrB7* AMR genes on an IncX3 plasmid (Fig. 2). The antibiotic resistance loci from the plasmid were found to be a composite transposon flanked by IS6-family elements inserted into the IncX3 backbone. However, the plasmid IncN from the Vellore isolate carried *bla*_TEM-1B_, *bla*_DHA-1_, *qnrB4*, and *sul1* resistance genes. Additionally, both *bla*_TEM-1B_ and *bla*_DHA-1_ carrying resistance region were located in different composite transposons flanked by IS6-family elements (Fig. 2). The IncX3 plasmid responsible for the cephalosporin resistance in *S.* Typhi in India is very closely related to the IncX3 plasmid in other Enterobacteriaceae.

## 3 Discussion

Currently, ceftriaxone and azithromycin are the drugs of choice and resistance to these agents challenges the treatment of typhoid fever. This has been demonstrated by the emergence of ceftriaxone resistant XDR *S*. Typhi in Pakistan, with resistance to five classes of antimicrobials, including all three first-line agents, fluoroquinolones and ceftriaxone. This study aimed to investigate the evolutionary insights that led to the emergence of cephalosporin resistant *S*. Typhi in India and its regional spread.

We identified five cephalosporin resistant *S*. Typhi isolates from India and characterized for their phylogenetic relationship with the global collection including the recently identified cephalosporin resistant *S.* Typhi from Bangladesh, Pakistan, and India. Our observations suggest that cephalosporin resistant *S*. Typhi isolates belonged to different lineages and carry various classes of β-lactamase gene in different plasmid backbones. Though all the study isolates of ceftriaxone resistant *S.* Typhi belong to H58 lineage II, the Vellore isolate (CMCST_CEPR_1: CP053702) were phylogenetically distant from the Mumbai isolates (Fig. 1). Hence, the emergence of Indian cephalosporin resistant *S*. Typhi appears to be *via* multiple independent genetic events and acquisition of different resistance plasmids carrying different β-lactamase genes. This is in line with the global emergence of cephalosporin resistance in *S*. Typhi as summarized in Table 1. So far, 18 studies have reported cephalosporin resistant *S.* Typhi across the globe, with the earliest report of *bla*_CTX-M-15_ gene in Western Asia during 2003–2006 [18]. While the majority of reports were based on phenotypic data, a few have reported genetic AMR and plasmid profiles. Except for the outbreak event reported from Pakistan [12], all other reports are of sporadic cases from different locations. Hence there is a lack of a global perspective of the evolution and spread of cephalosporin resistant *S*. Typhi.

In our study, the genomic characterization of cephalosporin resistant *S*. Typhi isolates from India showed that as in Pakistan, cephalosporin resistant isolates belong to the H58 lineage. However, within the H58 lineage, the cephalosporin resistant *S*. Typhi was distributed in different sub-lineages. The Indian isolates belonged to the dominant H58 lineage II while the isolates from Pakistan clustered with H58 lineage I. These results are in agreement with previous reports as the MDR associated lineage I is dominant in Pakistan and lineage II (QRDR triple mutants) is more prevalent in India [[10], [11], [12],^19^]. The only exception is the previously reported ceftriaxone resistant *S*. Typhi from Gurgaon, India, which originated from lineage H58 lineage I [17]. In contrast to the above observations, isolates from Bangladesh, Democratic Republic of Congo (DRC) and Philippines clustered with the non-H58 phylogenetic lineages [[20], [21], [22], [23]].

The evolutionary process of *S*. Typhi is driven by the emergence and spread of drug resistant lineages due to antibiotic usage selection pressure [10,24,25]. This is supported by earlier evolutionary events such as the integration of the MDR gene cassette (composite transposon) into the chromosome [26]*.* Further, the emergence and regional dominance of QRDR triple mutant lineage II in India was associated with high fluoroquinolone exposure in the region [7,11]. Based on available data, it is evident that H58 lineage I, with a chromosomal AMR cassette and single QRDR mutation (S83F), acquires and maintains plasmids harboring AMR determinants, as in the XDR Pakistan isolates [12]. Conversely, we note that H58 lineage II strains with double/triple QRDR mutations developed cephalosporin resistance by acquiring resistance plasmids such as IncX3 (*bla*_SHV-12_) or IncN (*bla*_TEM-1B,_ *bla*_DHA_) with no further spread documented. The exact sequential events of plasmid acquisition and maintenance by various *S*. Typhi lineages is poorly understood. Owing to these knowledge gaps, the limited spread of cephalosporin resistant H58 lineage II in India remains largely unexplained.

Historically, IncHI1 has been found to be strongly associated with multidrug resistance in *S*. Typhi. The association between *S*. Typhi and IncHI1 plasmid types was presumably due to the selective advantage of IncHI1 over the other circulating plasmids [27]. Consequently, the spread of MDR *S*. Typhi between the 1970s and 1990s was associated with independent acquisitions of IncHI1 plasmid types (PST) and in particular PST6 of IncHI1 in the global clonal expansion of H58 [10,27,28]. Similarly, in the Pakistan outbreak, a single acquisition event of IncY plasmid harboring *bla*_CTX-M-15_ led to the expansion of XDR *S*. Typhi (4.3.1.1P) with a difference of 6 SNPs from the MDR associated H58 *S*. Typhi [12]. In both cases the positive natural selection of the drug resistant lineages can possibly be attributed to either no measurable decrease in fitness after plasmid carriage or by compensatory evolution [29].

Together, the observations from our study re-emphasize that the dissemination of plasmid mediated AMR in clinical settings is based on the ability of bacteria to compensate for the initial fitness cost imposed by the plasmid acquisition [29]. Notably, IncY plasmid carrying *bla*_CTX-M-15_ acquired by predominantly circulating H58 lineage I in Pakistan is stable and well maintained likely due to the selective advantage carried by single QRDR mutation (S83F). However, the epidemiological question of why the predominant *S*. Typhi H58 lineage II harboring cephalosporin resistant gene is not widespread in India has been long unanswered. The data from our study suggests that this phenomenon may be the result of a negative fitness coefficient (MLE sˆ = −0.010) posed by the predominant highly fluoroquinolone-resistant QRDR triple mutant (S83F-D87N-S80I) confined to H58 lineage II circulating in India [30]. Even if this statement holds true for the QRDR triple mutants (study isolates), this could be one of the factors that explains the limited spread of these isolates. Unfortunately, data from previously published studies have not computed the fitness coefficients for S83F-E84K double mutants or E84K as a single mutant. In this context, the resultant fitness cost in double mutant isolate ‘CMCST_CEPR_1’ and single mutant isolate ‘Gurgaon02’ from India would also have to be explained through further studies. Alongside mutational impact, this would require investigation into the fitness effects of IncN plasmid carriage. Until then, it can only be hypothesized that the variation in acquisition of plasmids and its long-term persistence in *S*. Typhi is driven by multiple factors with host genotype being an important determinant [31].

Although limited in sample size and geographical representation, the preliminary observation derived from our study importantly highlights the ability of *S*. Typhi to acquire diverse plasmids to develop cephalosporin resistance. A follow up study on larger sample set, collected evenly across the endemic regions of South Asia, would be required to establish the rate of evolution and clonal expansion in these newly discovered cephalosporin resistant *S.* Typhi.

## 4 Materials and methods

### 4.1 Bacterial isolates, identification and AST

Five clinical isolates of ceftriaxone resistant *S.* Typhi from three different tertiary care hospitals in India between 2015 and 2018 were confirmed by serotyping according to the *Kauffmann-White* scheme [32] and standard microbiological techniques. Antimicrobial susceptibility testing (AST) was performed by using agar disk diffusion (DD) method for six antimicrobial agents including ampicillin (10 μg), chloramphenicol (30 μg), co-trimoxazole (1.25/23.75 μg), ciprofloxacin (5 μg), ceftriaxone (30 μg) and azithromycin (5 μg). Minimum inhibitory concentration (MIC) for ceftriaxone was determined using the broth micro dilution (BMD) method in accordance with the Clinical and Laboratory Standards Institute (CLSI) 2018 guidelines and interpretative criteria [33].

### 4.2 DNA extraction and whole genome sequencing

Genomic DNA of the study isolates was extracted from an overnight culture (14–16 h) grown at 37 °C on blood agar, using the QIAamp DNA Mini Kit (Qiagen, Hilden, Germany) according to the manufacturer's protocol. The extracted DNA was subjected to whole genome sequencing (WGS) using the Ion Torrent PGM sequencer (Life Technologies, Carlsbad, CA) with 400 bp read chemistry. Two of five isolates were sequenced using the Oxford Nanopore MinION sequencer (ONT, Oxford, UK) per standard protocol to fully resolve the plasmid structure.

### 4.3 Genome assembly, genotyping and plasmid typing

Hybrid genome assembly was carried out according to the standardized protocol developed in-house [34] using Unicycler hybrid assembly pipeline (v.0.4.6). Ion torrent reads assembly was achieved *de novo* in the SPAdes assembler (v.5.0.0.0) embedded in the Torrent suite server (v.5.0.3). The quality metrics of the assembled genome was analyzed using Quast (v.4.5) and genomes were annotated using Prokaryotic genome annotation pipeline (PGAP v.4.9) before being submitted to NCBI.

In-*silico* Multi-Locus Sequence Typing (MLST) was determined using Enterobase database (https://enterobase.warwick.ac.uk/species/index/senterica) available in pubMLST. The resistance profile of the assembled genomes was identified using ResFinderv.3.1 available from (https://cge.cbs.dtu.dk/services/ResFinder/). Isolates were genotyped using the genotyphi scheme as described on the GitHub repository (https://github.com/katholt/genotyphi). Plasmid typing was carried out using Plasmid finder available from the CGE server (https://cge.cbs.dtu.dk/services/PlasmidFinder/).

### 4.4 SNP calling and phylogenetic tree construction

Core genome single-nucleotide polymorphisms (SNPs) were identified using Snippy v.0.2.6 (https://github.com/tseemann/snippy) with CT18 (NC_003198) as the reference [35]. The recombination within the alignment file was filtered and removed using the Gubbins algorithm v. 2.0.0 [36] and the non-recombinant SNPs were used to construct the phylogenetic tree using Fast tree. The maximum - likelihood tree with 100 bootstrap values was rooted to the reference genome CT18 and labeled using the interactive tree of life software iTOL v.3 [37].

### 4.5 Plasmid characterization and comparative genomics

Plasmids from two representative isolates (One isolate each from Mumbai and Vellore) were circularized by combining the reads obtained from Ion torrent and MinION sequencing platforms as described earlier [34]. The remaining three IncX3 plasmids (carried by Mumbai isolates) were extracted from short reads using plasmidSPAdes [38] with representative plasmids as the reference. The plasmid comparison was visualized and analyzed using CGview server v.1.0 [39].

## 5 Conclusion

Even though ceftriaxone resistant *S.* Typhi are not widely seen in India at present, emergence and spread is possible due to the current high use of ceftriaxone for the treatment of typhoid fever. Hence, there are potential risks for the long-term persistence of the currently acquired plasmid or emergence of a more competitive plasmid in ceftriaxone resistant *S*. Typhi. This is based on the following evidence, (i) endemicity of *bla*_SHV_ and *bla*_CTX-M-15_ carrying plasmids in *E. coli* and *Klebsiella* sp. in India favoring horizontal gene transfer to *S*. Typhi, (ii) high use of ceftriaxone for the management of complicated typhoid fever posing antibiotic pressure, and (iii) the dominant H58 lineage in India being capable of acquiring plasmid harboring AMR determinants. Considering the prolonged maintenance of newly acquired *bla*_CTX-M-15_ carrying IncY plasmid in 4.3.1 lineage I in Pakistan, any trigger could possibly lead to similar events in India. This forewarns us that H58 lineage II are capable of acquiring MDR plasmids from other Enterobacteriaceae and could potentially cause a large outbreak. Hence, monitoring of cephalosporin resistant *S*. Typhi and its lineages associated with plasmid acquisition is required for early detection, in conjunction with rational use of antibiotics and prevention strategies for control of enteric fever.

## Declaration of Competing Interest

The authors declare that there is no potential conflict of interest.

## Appendix A Supplementary data

Supplementary Table 1 Image 1
